# Endoscopic resolution of esophageal varices in a patient with IgG4-related disease: a case report

**DOI:** 10.3389/fmed.2026.1926636

**Published:** 2026-09-28

**Authors:** Daniela Pinzon-Leal, Ivan Sisa, Fuatd Terán Misle

**Affiliations:** School of Medicine, Universidad San Francisco de Quito USFQ, Quito, Ecuador

**Keywords:** autoimmune pancreatitis, case report, esophageal varices, IgG4-related disease, portal hypertension, rituximab

## Abstract

IgG4-related pancreatitis is a complex and multiorgan disease that can mimic pancreatic malignancy. Corticosteroids are the first-line therapy, but their use may not be an option for patients with metabolic comorbidities, such as diabetes mellitus. We report a rare case of a 58-year-old man who presented with portal hypertension and esophageal varices. The patient underwent pancreaticoduodenectomy for suspected pancreatic malignancy. However, a surgical pathology report retrieved during revision confirmed IgG4-associated autoimmune pancreatitis and documented diffuse sclerosing lymphoplasmacytic inflammation with dense periductal lymphoplasmacytic infiltrates, obliterative phlebitis, fibrosis/sclerosis, and increased IgG4-positive plasma cells (up to >50 per high-power field). Serum IgG4 was recorded as 5,521 mg/dL. Postoperatively, the patient developed insulin-dependent diabetes mellitus, precluding corticosteroid therapy. Rituximab was initiated and the dosing interval was subsequently shortened after PET findings were interpreted as possible systemic involvement. Following treatment, recorded serum IgG4 values progressively decreased to 17 mg/dL. Follow-up endoscopy in 2023, supported by paired endoscopic images, demonstrated no visible esophageal varices in the imaged segment. This case describes an unusual and clinically relevant temporal association. However, the available data do not establish resolution of portal hypertension or a causal relationship between rituximab and the endoscopic finding.

## 1 Introduction

Autoimmune pancreatitis (AIP) is a chronic fibro-inflammatory pancreatitis that is mediated by the immune system, is sensitive to corticosteroids, can mimic pancreatic cancer, and is characterized by lymphoplasmacytic infiltrate with IgG4 plasma cells, storiform fibrosis, and obliterative phlebitis (1, 2). AIP has two distinct phenotypes: type 1 (IgG4-related disease, often affecting multiple organs) and type 2 (a pancreas-specific disorder, associated with inflammatory bowel disease and low IgG4 levels) (2–4). Its pathophysiology involves Th2/Treg responses with production of profibrotic cytokines and proliferation of IgG4 plasmablasts, which promote fibrosis and ductal damage. In type 1, cholangitis, sialadenitis, or nephritis often coexist, while type 2 is more associated with inflammatory bowel disease (IBD) and granulocytic epithelial lesions (GELs) observed in the pancreatic ducts (1, 3–5). Epidemiologically, AIP accounts for ∼2% of chronic pancreatitis cases, with a prevalence of <1/100,000, a male predominance, and diagnosis in the seventh decade for type 1 (1, 3). The diagnosis is based on integrated criteria from clinical findings, serology (IgG4), imaging, histology, and response to steroids (1–3). Peripancreatic vascular involvement has also been described in type 1 AIP (6, 7). This study presents the case of a 58-year-old patient with type 1 AIP who presented with esophageal varices and ultrasound-reported portal hypertension of undetermined cause. Paired endoscopic images documented disappearance of the visible varices during follow-up after pancreaticoduodenectomy and rituximab treatment. Because dedicated vascular imaging and portal-pressure assessment were unavailable, causality cannot be established.

## 2 Case description

1. Patient information

The patient is a 58-year-old male with a prior right inguinal hernia repair and no family history of relevant diseases. In September 2020, he presented with band-like abdominal pain for three consecutive nights.

2. Clinical findings

During the initial evaluation, the physical examination revealed significant weight loss. No comprehensive hepatic assessment capable of excluding cirrhosis was available.

3. Timeline and diagnostic assessments

In October 2020 an upper gastrointestinal endoscopy was performed revealing esophageal varices. Subsequently, an ultrasound was reported as showing portal hypertension with no apparent cause. In November 2020, following a COVID-19 infection, he experienced weight loss of 25 kg and a gout attack (right ankle, uric acid recorded as 9.5; unit unavailable). In March 2021, due to persistent symptoms and weight loss, an abdominal CT scan was performed, revealing filiform stenosis of the pancreatic head, dilation of the proximal bile duct, pancreatic-head enlargement, and three adenopathies, without a clearly visualized mass. Laboratory findings were summarized as elevated bilirubin with a cholestatic pattern, along with a CA 19-9 value of 670 units/ml. Based on these findings, a diagnosis of pancreatic cancer was considered. A surgical pathology report retrieved during revision identifies the procedure as a Whipple pancreaticoduodenectomy and lists the specimen collection date as 29 March 2021; a separate detailed operative report remains unavailable. The pathology report documented diffuse sclerosing lymphoplasmacytic inflammation of the pancreas, consistent with IgG4-associated autoimmune pancreatitis. Characteristic findings included a dense periductal lymphoplasmacytic infiltrate, obliterative phlebitis highlighted by elastic stain, and fibrosis/sclerosis. Immunohistochemistry showed increased IgG4-positive plasma cells relative to IgG-positive cells, up to >50 per high-power field, in both the pancreas and gallbladder. The inflammatory process also involved the gallbladder. Low-grade pancreatic intraepithelial neoplasia was noted, 21 lymph nodes were benign, and no carcinoma was identified. STAT6 and ALK-1 immunostains were negative. A numeric IgG4/IgG ratio was not reported, and representative histology images were not available. A separately submitted left lateral liver lesion was diagnosed as hemangioma. The pathology report was issued on 6 April 2021. On 29 April 2021, serum IgG4 was recorded as 5,521 mg/dL. As a consequence of the surgery, the patient developed insulin-dependent diabetes mellitus and exocrinepancreatic insufficiency. In 2022, PET-CT findings were interpreted by the treating physician as possible extra-pancreatic involvement affecting the colon, liver, prostate, and salivary glands; the original PET report and images were unavailable (Figure 1).

4. Therapeutic interventions

**Figure 1:**
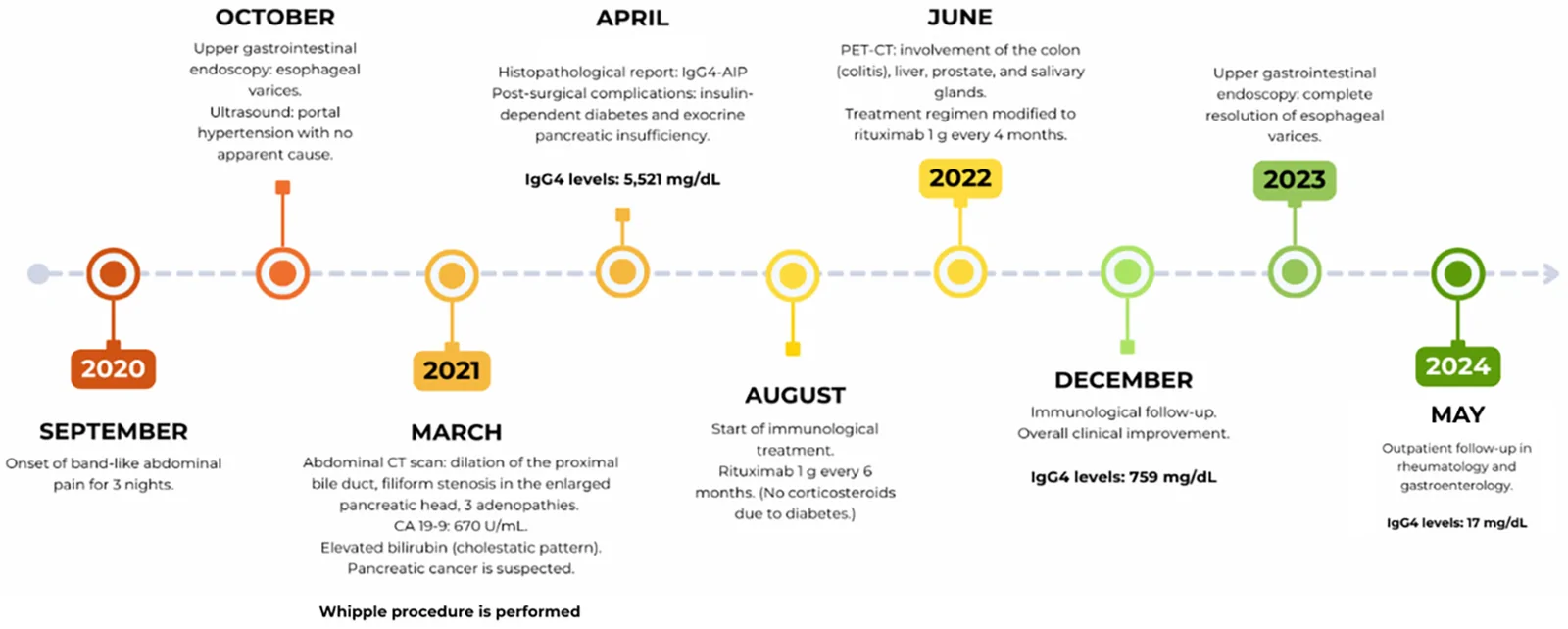
Timeline of diseasés course and therapeutic resolution of the case.

Given that the patient developed post-surgical insulin-dependent diabetes mellitus that was difficult to control, the use of corticosteroids as first-line treatment was avoided. Given the complexity of the case, rituximab was administered using an empirical regimen. In August 2021, treatment with 1 g of rituximab every 6 months was initiated. The available record did not specify whether each treatment cycle consisted of one or two infusions. In June 2022, after PET-CT findings were interpreted by the treating physician as possible extra-pancreatic involvement, the dosing interval was shortened to every 4 months. No additional clinical or biochemical reason for the interval change was documented. The patient also continued to be managed with basal and prandial insulin, glycemic monitoring with sensors, and pancreatic enzyme supplementation (two capsules of Creon 35,000 with each meal).

5. Follow-up and outcomes

The clinical course was favorable, with a progressive decrease in recorded serum IgG4 values: 5,521 mg/dL on 29 April 2021, 759 mg/dL on 20 December 2022, and 17 mg/dL on 21 May 2024 (Figure 1). In 2023, a follow-up upper gastrointestinal endoscopy, supported by the available image, documented no visible esophageal varices in the imaged segment (Figure 2). No follow-up Doppler ultrasound, dedicated splenoportal imaging, or portal-pressure measurement was available; therefore, this finding does not demonstrate resolution of portal hypertension.

**Figure 2:**
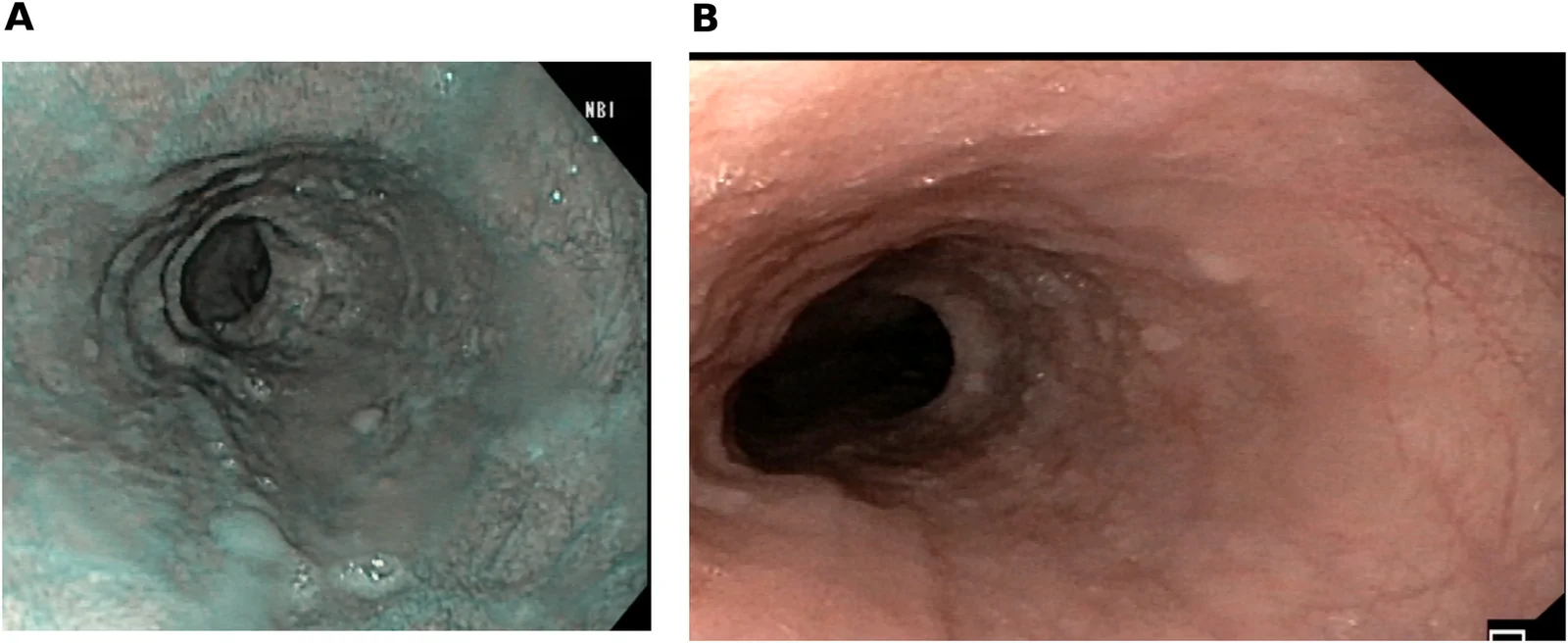
Endoscopic comparison. **(A)** shows the pretreatment upper gastrointestinal endoscopy in 2020 using narrow-band imaging, showing prominent longitudinal esophageal variceal columns. **(B)** shows the follow-up upper gastrointestinal endoscopy in 2023 using white-light imaging, showing no visible variceal columns in the imaged esophageal segment. The paired images document an endoscopic outcome but do not demonstrate normalization of portal pressure or identify the mechanism of the original portal hypertension.

## 3 Discussion

This study presents a case of type 1 autoimmune pancreatitis (IgG4-AIP) with esophageal varices that were no longer visible on follow-up endoscopy after pancreaticoduodenectomy and during rituximab treatment. This outcome is unusual and, to the best of our knowledge, has rarely been described in the literature. Thus, we consider it to be of clinical interest. However, the available evidence cannot establish that rituximab caused the endoscopic change or that portal hypertension resolved. The primary surgical pathology report, paired pretreatment and follow-up endoscopic images, and long-term follow-up are important strengths of the report.

However, the case also has limitations. Its anecdotal nature (single patient) prevents the establishment of causality and limits the generalizability of the results. Although a reduction in recorded serum IgG4 values was documented during rituximab treatment, the Whipple procedure performed previously may have modified portal hemodynamics, which does not rule out its influence on the evolution of the varices. Another limitation is the lack of histopathological confirmation of the reported extra-pancreatic manifestations. In addition, the detailed operative report, original laboratory reports, PET report and images, dedicated vascular imaging, and comprehensive hepatic assessment remained unavailable. The original surgical pathology report was successfully retrieved during revision; however, a numeric IgG4/IgG ratio and representative histology images were not available. These limitations prevent determination of the mechanism of portal hypertension and definitive exclusion of cirrhosis.

Type 1 autoimmune pancreatitis (IgG4-AIP) is a systemic fibro-inflammatory condition that can mimic pancreatic adenocarcinoma. It has a low prevalence, predominantly affects males, and is usually diagnosed in the sixth to seventh decade of life (1, 3). These characteristics match the profile of the patient presented, who was initially treated for probable pancreatic cancer and subsequently had IgG4-AIP confirmed in the surgical pathology report.

It has been documented that IgG4-related disease can spread beyond the pancreas, affecting the hepatobiliary, renal, prostatic, and salivary glands (3, 5). Furthermore, although vascular involvement is not a common manifestation, cases of peripancreatic vascular involvement secondary to AIP have been described. The pathophysiological explanation for this includes mechanical compression of adjacent vessels or extension of the fibroinflammatory process to vascular structures (6, 7). However, these mechanisms could not be confirmed in the present patient because dedicated vascular imaging was unavailable.

As for treatment, corticosteroids are the first line of treatment and usually induce remission in most patients (1, 3). However, the presence of insulin-dependent diabetes mellitus, as occurred in this case after Whipple surgery, may limit their use due to the risk of metabolic decompensation. In this context, rituximab has established itself as an effective therapeutic alternative in patients with contraindications to steroids or with refractory disease (3, 8, 9). It has been reported to induce and maintain clinical and biochemical remission in IgG4-related disease (8, 10); however, these data do not establish an effect on gastroesophageal varices.

Although there are multiple reports on the efficacy of rituximab in inducing and maintaining remission of IgG4-AIP, evidence regarding the resolution of associated vascular complications is limited. The present case reports an unusual finding: disappearance of visible esophageal varices during follow-up after pancreaticoduodenectomy and during rituximab treatment. This temporal sequence does not demonstrate an additional vascular benefit of rituximab. Available studies on corticosteroid treatment show variable results. While some authors have reported improvement in peripancreatic vascular lesions, others have described persistent gastric varices and splenic venous obstruction (7, 11, 12). More recent cases required direct endoscopic or surgical treatment of gastric varices (13, 14). A comparison of selected published cases is provided in Table 1.

**Table 1:** Selected published cases of autoimmune pancreatitis with gastroesophageal varices and comparison with the present case.

Report	Vascular/variceal findings	Treatment	Outcome
Goto et al. (11)	Splenic-vein obstruction and gastric varices	Glucocorticoids	Varices disappeared in one case and persisted in two
Juarez et al. (12)	Splenic- and superior-mesenteric-vein obstruction; gastric varices	Glucocorticoids/supportive care	Obstruction and varices persisted or recurred
Iwasa et al. (13)	Splenic vascular obstruction; bleeding gastric varices	Endoscopic treatment and splenectomy	Bleeding controlled after direct treatment
Xi et al. (14)	Splenic-vein occlusion; bleeding gastric varices	EUS-guided coil/tissue glue and rituximab	Varices improved after direct endoscopic therapy
Present case	Esophageal varices; mechanism unavailable	Pancreaticoduodenectomy and rituximab	No visible varices on 2023 endoscopy; causality unproven

Pancreaticoduodenectomy is an important potential confounder. Resection of an inflammatory pancreatic-head lesion could theoretically reduce local mass effect, whereas pancreaticoduodenectomy can also alter portal hemodynamics when the portal-mesenteric-splenic venous confluence is resected or when postoperative portal-vein stenosis develops (15, 16). Because the detailed operative report and serial vascular imaging were unavailable, the effect of surgery on portal flow cannot be determined in this patient.

A hepatic cause of portal hypertension also cannot be excluded. The available clinical summary did not include systematic liver morphology, platelet trends, liver stiffness, histology, or a complete longitudinal hepatic laboratory profile. The PET finding described as “liver involvement” could not be independently characterized because the original report and images were unavailable.

## 4 Conclusion

This case shows the multisystemic nature of IgG4-AIP and suggests the possible role of rituximab as an alternative to corticosteroids. Furthermore, it documents endoscopic disappearance of previously visible esophageal varices during follow-up after pancreaticoduodenectomy and rituximab treatment. Because the mechanism of portal hypertension and the relative contributions of surgery, immunotherapy, and other factors could not be determined, the finding should be interpreted as a temporal association rather than proof of causality. Further reports with dedicated vascular imaging and systematic hepatic assessment are required.

## Data Availability

The original contributions presented in the study are included in the article/Supplementary Material, further inquiries can be directed to the corresponding author.
